# Multisystem immune-related adverse events due to toripalimab: Two cases-based review

**DOI:** 10.3389/fcvm.2022.1036603

**Published:** 2022-11-25

**Authors:** Yanran Chen, Yulan Chen, Jingyi Xie, Dongzhou Liu, Xiaoping Hong

**Affiliations:** ^1^The Second Clinical Medical College, Jinan University, Shenzhen, China; ^2^Department of Rheumatology and Immunology, Shenzhen People’s Hospital, The Second Clinical Medical College, Jinan University, Shenzhen, China; ^3^The First Affiliated Hospital, Southern University of Science and Technology, Shenzhen, China

**Keywords:** immune checkpoint inhibitors, PD-1, immune-related adverse events, myocarditis, pneumonia

## Abstract

Immune checkpoint inhibitors (ICIs) have significantly improved the survival of patients with advanced tumors. However, immune-related adverse events (irAEs) caused by ICIs, especially high-grade irAEs, are of growing concern. High-grade multisystem irAEs due to toripalimab, a programmed cell death-1 (PD-1) inhibitor, have been rarely reported. Two patients with malignant metastatic tumors were treated with anti-PD-1 immunotherapy. However, both patients developed high-grade multisystem irAEs based on myocarditis, with chest discomfort and malaise as the main clinical manifestation. Both patients had an elevation of cardiac enzymes, abnormal electrocardiography and left ventricular wall motion. Patient 2 was also diagnosed with organizing pneumonia. Immunotherapy was suspended. High-dose intravenous methylprednisolone was immediately initiated. The patients’ symptoms were significantly relieved in a short period of time. Immunosuppressants were discontinued at the 6th month follow-up in patient 1 without relapse. However, patient 2 was lost to follow up due to financial reasons. To the best of our knowledge, this is the first report regarding ICI-associated myocarditis-pneumonia due to toripalimab, indicating the significance of early recognition and management of high-grade multisystem irAEs in clinical practice.

## Introduction

Immune checkpoint inhibitors (ICIs) have dramatically extended the survival of patients with advanced tumors. However, immune-related adverse events (irAEs) caused by ICIs, especially high-grade irAEs, pose a significant threat to patients’ lives, with an incidence ranging from 54 to 76% (1). Excessive immune activation by ICIs can occasionally induce multiple irAEs in different organs (2). The incidence of multisystem high-level irAEs may be underestimated due to the high rate of misdiagnosis that may result from its individualized clinical presentation.

Toripalimab, a human monoclonal antibody against programmed cell death-1 (PD-1), has been developed and received conditional approval as salvage treatment for unresectable or metastatic melanoma in China since 2018 (3). Yet high-grade multisystem irAEs caused by toripalimab have been rarely reported. Here we report two Chinese patients with advanced tumors who experienced a severe storm of multisystem irAEs after receiving toripalimab treatment, indicating the significance of early diagnosis and timely management of multisystem irAEs due to toripalimab.

## Case presentation

### Case 1

A 43 years-old Chinese female patient had a 6-year history of mixed liposarcoma of the right upper extremity with bilateral lower extremity metastases. The patient received 240 mg of toripalimab every 2 weeks from May 2019. After four cycles of toripalimab treatment, she presented to the emergency department at our institution with complaints of a 5-day generalized rash and malaise, as well as a sudden onset of severe chest pain with palpitation and dyspnea lasting for 2 h. She denied a history of diabetes mellitus, cardiovascular diseases, or thyroid diseases. Blood pressure on admission was 101/58 mmHg and the heart rate was irregular with a frequency of 131 beats/min. The respiratory rate was 36 breaths/min with normal oxygen saturation. Physical examination revealed generalized multiforme-like rash as well as muffled heart sounds and a grade II/VI holosystolic murmur typical of mitral regurgitation. Neurological examination revealed a grade IV muscle strength in her both lower limbs.

Laboratory tests showed that serum creatine phosphokinase (CPK) was 2,370 U/L (normal 25∼192 U/L), with increased levels of creatine kinase isoenzyme MB (CK-MB 53.6 ng/ml, normal 2∼4.99 ng/ml), cardiac troponin I (cTnI 7.49 ng/ml, normal 0∼0.02 ng/ml), cardiac troponin T (cTnT 5.43 ng/ml, normal 0∼0.014 ng/ml), myoglobin (214 ng/ml, normal 0∼46.6 ng/ml), lactate dehydrogenase (LDH 616 U/L, normal 110∼240 U/L), alanine aminotransferase (ALT 73 U/L, normal 0–40 U/L), aspartate aminotransferase (AST 212 U/L, normal 0–45 U/L), and N-terminal pro brain natriuretic peptide (NT-proBNP 4999 pg/ml, normal 0∼450 pg/ml). Further tests revealed decreased levels of free triiodothyronine (2.44 pmol/L, normal 3.28∼6.47 pmol/L) and free thyroxine (3.72 pmol/L, normal 7.64∼11.3 pmol/L), with elevated levels of thyrotropin (68.29 mIU/L, normal 0.38∼5.91 mIU/L), anti-thyroid peroxidase antibodies (94.99 mIU/ml, normal 0∼34 mIU/ml), and anti-thyroglobulin antibodies (572 IU/ml, normal 0∼115 IU/ml) (Table 1). There was no evidence of infection, and her autoimmune antibodies, including myositis-associated and myositis-specific antibodies, were also negative. Electrocardiogram showed paroxysmal ventricular tachycardia (104 beats/min). Emergency coronary angiography and left heart catheterization were unremarkable. Echocardiography revealed the myocardial motion of the anterior wall and the apical segment of the anterior septum of the left ventricle was diminished. The left atrium and left ventricle were enlarged (anterior-posterior left atrial diameter was 38 mm). In contrast, the right atrium and right ventricle were normal, with moderate mitral and tricuspid regurgitation and a small amount of pericardial effusion, with an ejection fraction (EF) of 55%. Electromyography demonstrated varying degrees of myogenic damage in the proximal muscles of the extremities, without abnormal changes in the distal muscles. Thyroid ultrasound and chest computed tomography (CT) scan showed no abnormalities. Cardiac magnetic resonance (MR) and muscle biopsy was not allowed due to the rapid deterioration of chest pain. She was transferred to the intensive care unit.

**TABLE 1:** Laboratory findings on admission.

Parameter	Case 1	Case 2	Reference range
Leukocytes (10^9^/L)	5.83	7.56	04–10
Erythrocytes (10^12^/L)	2.9	3.44	3.5–5
Hemoglobin (g/L)	90	102	110–150
Platelets (10^9^/L)	98	401	100–300
CPK (U/L)	2,370	1,365	25–192
CK–MB (ng/ml)	53.6	21.32	2–4.99
cTnI (ng/ml)	7.49	0.24	0–0.02
cTnI (ng/ml)	5.43	1.872	0–0.014
ALT (U/L)	73	14.8	0–45
AST (U/L)	212	27.6	0–40
Albumin (g/L)	36.3	31.2	35–55
IgG (g/L)	8.01	7.16	8–18
ALP (U/L)	68	71	15–121
Creatinie (μmol/L)	103	56	44–133
Urea nitrogen (mmol/L)	3.65	6	2.5–7.5
CRP (mg/L)	3.8	4.37	<*5*
PCT (ng/ml)	<0.05	<0.05	<0.05
ANA (AU/ml)	<32	2152	<32
Anti-dsDNA	Negative	Negative	Negative
pANCA	Negative	Negative	Negative
cANCA	Negative	Negative	Negative
Anti-Ro60	Negative	Positive	Negative
Anti-Ro52	Negative	Positive	Negative
Anti-SS-B	Negative	Negative	Negative
Anti-U1-snRNP	Negative	Positive	Negative

CPK, creatine phosphokinase; CK-MB, creatine kinase-MB; cTnI, cardiac troponin I; cTnT, cardiac troponin T; ALT, alanine aminotransferase; AST, aspartate aminotransferase; IgG, immunoglobulin G; ALP, alkaline phosphatase; CRP, C-reactive protein; PCT, procalcitonin; ANA, anti-nuclear antibodies; Anti-dsDNA, anti-double-stranded (ds) DNA antibody; pANCA, perinuclear anti-neutrophil cytoplasmic antibody; cANCA, cytoplasmic anti-neutrophil cytoplasmic antibody; Anti-SS-B, anti-Sjögren’s syndrome antigen B antibody; Anti-U1-snRNP, anti-U1-small nuclear ribonucleic particle antibody.

**FIGURE 1:**
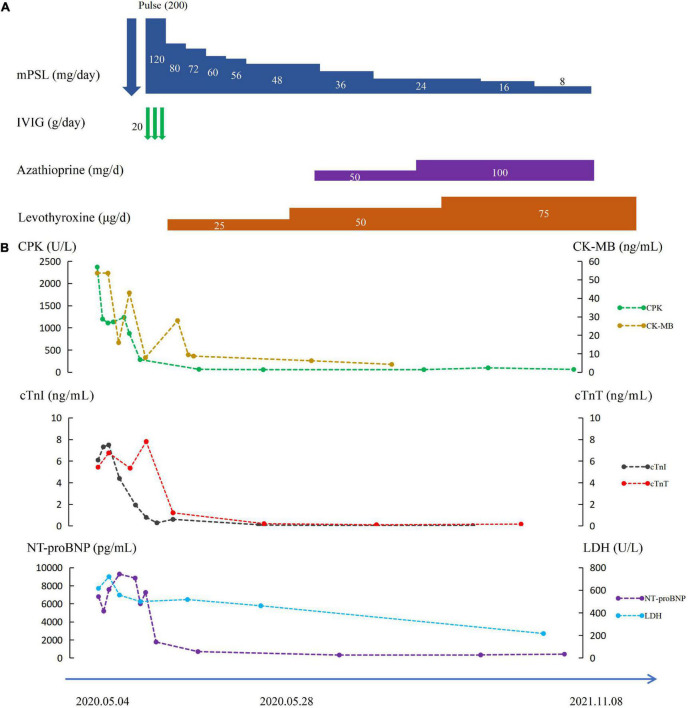
**(A)** Electrocardiogram of patient 1 on admission. **(B)** Clinical course of patient.

The patient was finally diagnosed with multisystem irAEs resulting from anti-PD-1 therapy according to the results of the multidisciplinary discussion. The diagnoses of ICI-associated myocarditis (grade 4), myositis (grade 3), Hashimoto’s thyroiditis (grade 2), and skin toxicity (grade 2) were made based on the National Cancer Institute Common Toxicity Criteria for Adverse Events (NCI-CTCAE). On day 3, intravenous methylprednisolone (200 mg/day) was initiated for 3 days, with oral levothyroxine replacement therapy (50 μg/day). Her chest pain and dyspnea were significantly relieved and the rash disappeared within 10 days. CPK, CK-MB, and troponin gradually decreased (Figure 1). On day 7, laboratory tests showed serum CPK to be 284 U/L, cTnI 0.279 ng/ml, cTnT 1.367 ng/ml, myoglobin 34 ng/ml, CK-MB 7.91 ng/ml, and NT-proBNP 1792 pg/ml. Echocardiography suggested an increase in EF to 60%.

Her methylprednisolone was gradually tapered. After 3 weeks, methylprednisolone was reduced to 48 mg/day, and she again developed chest pain and malaise. Laboratory tests showed a normal CPK level but an elevated CK-MB level to 28.7 ng/ml. Therefore, azathioprine (50 mg/day) was added, and her symptoms were gradually relieved within 2 weeks. Methylprednisolone was gradually tapered accordingly and was discontinued after 12 weeks, with azathioprine (100 mg/day) as maintenance therapy. At 6-month follow-up, laboratory tests revealed CPK to be 212 U/L, CK-MB 3.84 ng/ml, CTnI 0.08 ng/ml, and CTnT 0.016 ng/ml (Figure 1). At the time of writing this report, she was only taking oral levothyroxine (75 μg/day) replacement therapy and refused to receive any other anti-PD-1 therapies due to the potential risk for recurrence of severe multisystem irAEs, without evidence of tumor progression.

### Case 2

A 64 years-old Chinese woman was diagnosed with advanced pancreatic adenocarcinoma with multiple metastases (liver, adrenal glands, and multiple lymph nodes) in January 2021. Due to her intolerance to chemotherapy, toripalimab (240 mg every 2 weeks) was initiated and clinical improvement was gradually achieved. Before the sixth treatment, she developed chest tightness and malaise that lasted for 2 days, without fever or cough. She took amlodipine for 7 years due to hypertension, and she denied prior history of lung diseases or autoimmune diseases. Physical examination on admission showed that the patient had an oxygen saturation of 87% (room air, at rest) and wet rales in the right lower lungs, with normal cardiac auscultation.

**FIGURE 2:**
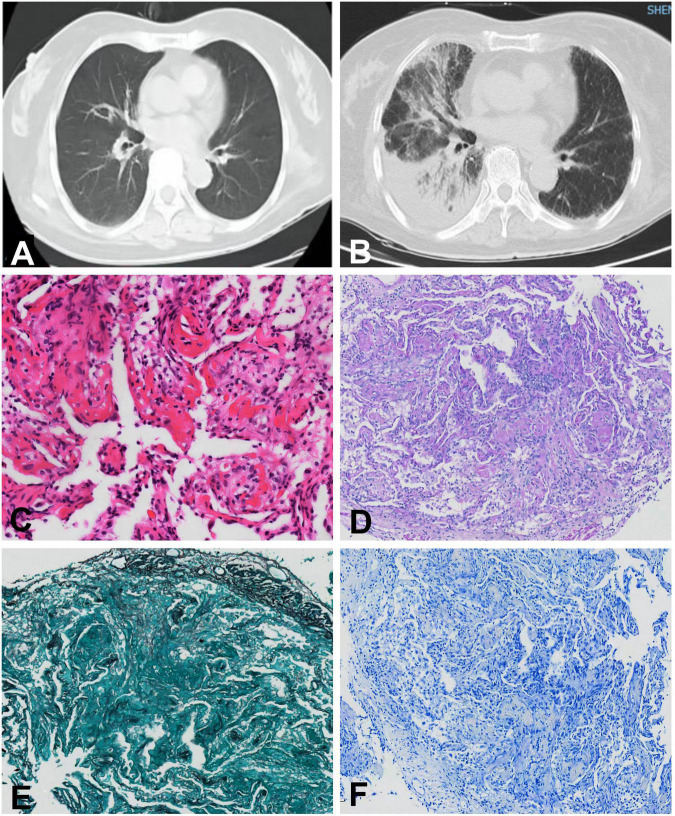
Chest computed tomography and histopathological changes of the lung biopsy in case 2. Chest computed tomography before **(A)** and after **(B)** onset of pneumonia. **(C)** Hematoxylin-eosin staining (×20). **(D)** Periodic acid-Schiff staining (×10). **(E)** Hexosamine silver staining (×10). **(F)** Antacid staining (×10).

Laboratory tests showed that serum CPK was 1322 U/L, CK-MB 101.2 ng/ml, CTnI 0.72 ng/ml, CTnT 1.872 ng/ml, myoglobin 146.3 ng/ml, and NT-proBNP 3964.8 pg/ml. The white blood cell count was 9.18 × 10^9^/L (normal 4∼10 × 10^9^/L), and calcitonin (<0.05 ng/ml, normal <0.05 ng/ml) and C-reactive protein (2.73 mg/L, normal <5 mg/L) levels were normal. Arterial blood gas analysis showed an oxygenation index of 281. Further examination revealed positive anti-nuclear antibodies (ANA, 2152 AU/ml, normal 0∼32 AU/ml) by chemiluminescent immunoassay and positive anti-U1-small nuclear ribonucleic particles (snRNP), anti-Ro60, and anti-Ro52 antibodies by line immunoassay (Table 1). Her electrocardiogram showed a complete right bundle branch conduction block. The echocardiogram showed reduced anterior interventricular wall segmental motion in the left ventricle with an EF of 49%. Chest CT of the chest showed consolidative opacities predominantly in the right basal parenchyma and a small amount of right pleural fluid. Lymphocyte subpopulation testing of the patient’s bronchoalveolar lavage fluid showed that CD3^+^ T cells accounted for 95.8% of lymphocytes, without evidence of infection. Lung biopsy showed alveolar wall edema suggestive of alveolitis, with a large amount of fibrinous exudate and lymphocytic infiltrate in the alveolar space, with no hyaline membrane formation (Figure 2). No malignant cells were seen in the patient’s bronchoalveolar lavage fluid or lung tissue biopsy.

The patient was diagnosed with ICI-associated myocarditis (grade 3) and ICI-associated pneumonia (grade 3) based on NCI CTCAE. Intravenous methylprednisolone (200 mg/day) was started on the second day of admission, along with intravenous immunoglobulin (20 g/day) for 3 days. The patient’s chest tightness improved and the oxygenation index increased to 323. On day 4 of admission, the patient was discharged without a repeated CT scan according to her wish.

## Discussion

Immune checkpoint inhibitors such as those targeting PD-1, PD-1 ligand 1 (PD-L1), and cytotoxic T lymphocyte-associated antigen 4 (CTLA-4) have been the most significant breakthroughs in cancer immunotherapy in recent years. These ICIs enhance immune surveillance and reduce the immune escape of cancer cells by “releasing the brakes” on the T-cell activation pathway, which may affect multiple organs. The NCI-CTCAE classifies irAEs into five levels, from mild, moderate, severe, life-threatening, to fatal (4). The most common fatal irAEs include myocarditis, pneumonia, encephalitis, and fulminant hepatitis, with a mortality ranging from 0.3 to 1.3% (2). Toripalimab is a relatively new ICI developed in China and was generally well-tolerated in clinical trials in Chinese patients with advanced malignancies. The high-grade irAEs (grade 3 or higher) associated with toripalimab was most common in the hematologic and hepatic system (3). Multisystem irAEs and fatal irAEs, such as myocarditis and pneumonia, caused by toripalimab have been rarely reported. Here we report two Chinese patients with advanced tumors who suffered from fatal multisystem irAEs after receiving toripalimab treatment. To the best of our knowledge, this is the first study describing a patient who concurrently experienced ICI-associated myocarditis and pneumonia, which indicates the significance of early recognition and management of multisystem irAEs due to toripalimab.

Cardiac immune-related events include the development of myocarditis, pericarditis, pericardial effusion, arrhythmias, myocardial infarction, and heart failure. The mechanisms by which ICIs cause myocarditis are unclear. Most of the existing studies have been attributed to the presence of shared antigens between the tumor and myocardium (5). Several studies have confirmed that PD-1 is found in the myocardium of patients with ICI-associated myocarditis with expansion of T cell clones. T cell receptors bind to homologous muscle antigens and tumor antigens, causing damage similar to viral myocarditis (5, 6). Even though ICI-associated myocarditis only seems to account for about 2.4% of all irAEs (5, 7), its mortality rate may reach 50% (8). The first 3 months of receiving ICIs are considered as a high-risk period for developing immune myocarditis (7–9). Mahmood et al. (7) showed that the median time from the first exposure to ICIs to the onset of myocarditis was 34 days. Remarkably, cases of immune myocarditis that received combination immunotherapy occurred even after the first receipt (10). PD-1/PD-L1 inhibitors are more likely to cause myocarditis and pericardial disease than CTLA-4 inhibitors (11). Accordingly, both patients in this study developed myocarditis within the first 3 months of receiving toripalimab, suggesting an extra attention should be paid during the high-risk period after toripalimab treatment. Therefore, informing patients receiving ICIs for the first time is recommended to pay special attention to symptoms such as chest pain and palpitations and to seek prompt medical attention. For patients with a history of ICIs and symptoms such as chest tightness, chest pain, and weakness, clinicians should consider ICIs-cardiomyopathy and perform electrocardiography, cardiac enzymology, and echocardiography.

The initial symptoms of ICI-associated myocarditis are heterogeneous and may include vague manifestations such as discomfort, fatigue, and weakness associated with the primary diseases, which are difficult to distinguish and can be easily overlooked. Almost all patients with myocarditis had elevated troponin and CPK levels, and 89% of cases showed electrocardiographic arrhythmias, including atrial fibrillation, premature ventricular beats, conduction block, and ventricular tachycardia. Cardiac ultrasound was suggested as a baseline assessment tool because about three-fourth of the patients showed abnormal left ventricular EF values after the onset of the disease (12). Enhancement of cardiac MR is an important modality in the available non-invasive diagnosis of myocarditis and 48% of patients with myocarditis may demonstrate late gadolinium enhancement (13). Endomyocardial biopsy is the gold standard for the diagnosis of myocarditis, which is characterized by myocardial infiltrates, including CD4 and CD8-positive T lymphocytes as well as macrophages (14). However, myocardial biopsy has its technical limitations, especially in cases of patchy or focal myocardial infarction-associated myocarditis (15). Furthermore, coronary angiography can help make a differential diagnosis in some cases with myocarditis that mimic coronary artery diseases, as demonstrated in patient 1 in this study. Most patients with clinically advanced tumors refuse to undergo this invasive procedure after a difficult tumor identification and treatment. Enhanced MR is considered an important non-invasive tool for diagnosing myocarditis, but patients prefer to spend their limited treatment costs on anti-tumor drugs due to their high price. In addition, although cardiac MR and endomyocardial biopsy was not performed in these two patients, myocardial abnormalities, such as significantly elevated levels of myocardial enzymes, ventricular dysfunction and arrhythmias, strongly supported the diagnosis of myocarditis.

Shao et al. reported an overall incidence of 4.5% for ICI-associated pneumonia (16). The incidence of severe pneumonia (grade 3 or higher) was reported to be 0.8–1.5% (17). Studies have shown that lung cancer patients treated with PD-1 or PD-L1 inhibitors were more likely to develop pneumonia compared to CTLA-4 inhibitors (2.7 vs. 1%) (17–19). ICI-associated pneumonia mainly includes organizing pneumonia (OP), non-specific interstitial pneumonia, hypersensitivity pneumonia, diffuse alveolar injury, acute interstitial pneumonia, and acute respiratory distress syndrome (20–22). OP was the predominant type of ICI-associated pneumonia, accounting for 77.8% of cases (22). Bronchoalveolar lavage and transbronchial lung biopsy were recommended in patients with ICI-associated pneumonia (14, 23). Based on Naidoo et al. criteria for ICI-associated pneumonia (24), the diagnosis of OP (grade 3) can be made in case 2. In addition, the incidence of thyroid dysfunction due to anti-PD-1 antibody treatment (5–10%) is higher than that after CTLA-4 inhibitors (0–5%) (25–27). Thyroiditis caused by ICIs usually causes transient hyperthyroidism, which often progresses to hypothyroidism. Notably, in this study, the diagnoses of myocarditis, myositis, Hashimoto’s thyroiditis, and skin toxicity were concurrently made in patient 1, and ICI-associated myocarditis and pneumonia was made in patient 2, which indicates multisystem irAEs in both of these two patients.

In a clinical study including 623 patients, 5.8% of patients experienced multisystem irAEs due to ICIs, with the combination of hepatitis-thyroiditis (10%) and dermatitis-pneumonia (10%) being the most common (28). ICI-associated myocarditis was most commonly accompanied by myositis (29%) and hepatitis (21%), followed by thyroiditis (12%) (9). It has been shown that myositis was the second frequent rheumatic and musculoskeletal irAEs (accounting for 36.1%), in which the mortality was 24% for myositis and 56.7% for concurrent myocarditis (29). The mechanism of myocarditis-myositis is unclear. Still, the theory of shared antigens between myocardium, skeletal muscle, and tumors has also been highlighted in the previous studies (6, 29). Importantly, myocarditis with myositis and/or myasthenia gravis overlap syndrome was reported to have a 60% mortality rate and is a hot issue in ICI-associated cardiomyopathy involving multisystem irAEs (30, 31). Therefore, it is reasonable to speculate that patients with multisystem irAEs based on myocarditis could have a higher mortality. Early recognition and diagnosis of multisystem irAEs are pivotal in the management of patients undergoing ICI treatment. Multisystem irAEs based on myocarditis reported previously are summarized in Table 2. Furthermore, to our knowledge, case 2 is the first report of myocarditis-pneumonia caused by toripalimab.

**TABLE 2:** Case reports regarding multisystem immune-related adverse events (irAEs) based on myocarditis induced by programmed cell death-1 (PD-1) inhibitors.

Case	Gender/ Age	Tumor	PD-1 inhibitor	Time^#^	Symptom	irAE	Treatment	Outcome
1 (36)	M/78	Melanoma	Pembrolizumab	5	Limb weakness	Myocarditis, myositis	PSL, PLEX	Dead due to respiratory failure
2 (37)	M/80	Melanoma	Nivolumab	2	Anorexia	IM3OS	PSL, IVIG, IA, PLEX	CPK levels normalized within 4 months
3 (38)	M/63	Melanoma	Nivolumab	2	Muscle pain	Myocarditis, myositis	PSL	Dead due to heart failure
4 (39)	F/55	Melanoma	Nivolumab	4	Ophthalmoplegia	IM3OS	PSL, IVIG, IA, PLEX	Weaned off ventilatory support at the 6 months
5 (40)	F/47	Melanoma	Toripalimab	4	Diplopia	IM3OS	PSL, IVIG, ventilator, PM	CPK levels normalized within 2 months
6 (41)	M/75	LUAD	Pembrolizumab	3	Asthenia	IM3OS, hepatitis, pneumonia	PSL, IVIG, IA, PLEX, IFX, ventilator	Dead due to respiratory failure
7 (42)	F/53	Thymoma	Pembrolizumab	4	Cough	Myocarditis, MG, liver and kidney dysfunction	PSL, IVIG, euthyrox, pyridostigmine	CPK levels normalized within 6 months
8 (43)	M/66	Thymoma, LUAD	Sintilimab	3	Fatigue, myalgia	Myocarditis, MG	PSL, IVIG, PLEX, euthyrox, pyridostigmine	CPK levels normalized within 3 months
9 (44)	F/48	Thymoma	Pembrolizumab	2	Shortness of breath	Myocarditis, MG	PSL, IVIG, IFX, PM, ventilator, ECMO	Dead due to heart failure
10 (45)	F/45	Thymoma	Pembrolizumab	2	Quadriparesis	Myocarditis, MG, liver dysfunction	PSL, IVIG, IA, ventilator, PM, ECMO	Dead due to hypoxic brain damage

^#^Time between initiation of PD-1 inhibitors and irAE (weeks). PSL, prednisolone; PLEX, plasma exchange; IM3OS, myocarditis with myositis and myasthenia gravis overlap syndrome; IVIG, intravenous immunoglobulin; IA, immunoadsorption; PM, pacemaker; LUAD, lung adenocarcinoma; IFX, infliximab; ECMO, extracorporeal membrane oxygenation; MG, myasthenia gravis.

Low-grade irAEs respond rapidly to steroid therapy and generally do not result in hospitalization or termination of treatment with ICIs. Permanent discontinuation of ICIs is recommended for patients with severe myositis or myocarditis and myositis-carditis overlap (32). Patients with ICI-associated myocarditis required immediate initiation of intravenous corticosteroid therapy and consideration of escalation to immunoglobulin, cyclophosphamide, rituximab, azathioprine, or methotrexate if patients do not respond well to corticosteroid alone (4, 14). Infliximab is the recommended second-line agent for ICI-associated myocarditis. Both abciximab and tacrolimus have been reported (1, 33), and tofacitinib has recently been reported to have significant efficacy in immune-associated myocarditis (34). Notwithstanding, even based on the above treatment, the mortality rate of myocarditis still reaches 50% (35). Hence, an early recognition and intervention of myocarditis is beneficial to improve the prognosis. Notably, both patients in this study had a positive response to glucocorticoids. Patient 1 experienced a recurrence of symptoms during glucocorticoid tapering, and a positive effect was obtained with the addition of azathioprine. In case 2, the patient also showed improvement after methylprednisolone and immunoglobulin therapy.

## Conclusion

In conclusion, we described two Chinese patients with advanced tumors suffering from severe multisystem irAEs based on after receiving toripalimab treatment. Improved awareness and an early identification of multisystem irAEs are of great importance in management of patients undergoing treatment of ICIs.

## Data availability statement

The original contributions presented in this study are included in the article/supplementary material, further inquiries can be directed to the corresponding author.

## Ethics statement

Written informed consent was obtained from the individual(s) for the publication of any potentially identifiable images or data included in this article.

## Author contributions

XH initiated and reviewed the manuscript. YRC and YLC were involved in the data collection and writing of the manuscript. JX provided the clinical details. DL was responsible for steering the direction of the manuscript. All authors read and approved the final manuscript for submission.
